# Laparoscopic Cystectomy for a 20-Centimetre Ovarian Endometrioma in a Subfertile Patient: Α Case Report

**DOI:** 10.7759/cureus.54386

**Published:** 2024-02-18

**Authors:** Georgios Grigoriadis, Alexandros Lazaridis, Andres Vigueras Smith, Angelos Daniilidis

**Affiliations:** 1 Centre for Endometriosis, Saint Luke’s Hospital, Thessaloniki, GRC; 2 Department of Obstetrics and Gynaecology, Aretaieion University Hospital, Athens, GRC; 3 Department of Obstetrics and Gynaecology, Hospital las Higueras, University of Concepcion, Talcahuano, CHL; 4 1st University Department in Obstetrics and Gynecology, Papageorgiou General Hospital, School of Medicine, Aristotle University of Thessaloniki, Thessaloniki, GRC

**Keywords:** case report, cystectomy, laparoscopy, ovarian endometrioma, endometriosis

## Abstract

Large ovarian endometriomas may cause severe pressure symptoms and often require surgical management. The laparoscopic approach, although challenging, is feasible and safe when performed by surgeons with advanced minimal access skills, provided that certain rules are respected. We report a case of a 40-year-old, nulliparous patient with a history of endometriosis, low ovarian reserve, and subfertility who presented with a 20-cm left ovarian endometrioma and associated symptoms, managed successfully by laparoscopic cystectomy. Compared to non-excisional surgical methods, endometrioma cystectomy likely causes a more profound decline in post-operative ovarian reserve, which is particularly important in the context of subfertility. We discuss the technical aspects of this challenging procedure, potential alternative approaches, and clinical decision-making as to why cystectomy was preferred.

## Introduction

Endometriosis is a benign, estrogen-dependent gynaecological disease that affects 20-50% of the subfertile female population [1]. Endometrioma (OMA) is one of the three recognized phenotypes of endometriosis, with the other two being superficial peritoneal endometriosis (SUP) and deep endometriosis (DE) [2]. The presence of OMA per se reduces ovarian reserve [3]; however, its surgical management is likely to accentuate the decline [4]. Although various methods of surgical management of endometrioma exist [4], cystectomy remains the most commonly performed technique, associated with higher spontaneous conception rates and lower risk of recurrence [5]. However, this surgical approach may cause a higher drop in ovarian reserve compared to non-excisional methods [6]. Large OMAs extending above the pelvis are uncommon, and their laparoscopic management is, often, technically challenging.

In this case report, written according to the CARE Checklist on writing case reports [7], we describe the laparoscopic management of a 20-cm ovarian endometrioma by cystectomy in a 40-year-old, symptomatic, nulliparous patient with subfertility. We discuss the technical aspects of this challenging operation, its implications on fertility, as well as potential alternative surgical approaches to OMA cystectomy.

## Case presentation

A 40-year-old, nulliparous patient attended the outpatient specialist endometriosis clinic with a history of endometriosis, pelvic pain, and severe pressure symptoms. She had not attended routine gynaecological screening in the last three years. Her past surgical history included one laparotomy (through Pfannenstiel incision) for a 10-cm right OMA cystectomy (2007), and two laparoscopies: one cystectomy for a 5-cm left OMA (2013) and one right salpingectomy for right hydrosalpinx (2017). During the second laparoscopy (2017), a left hydrosalpinx was also identified; however, due to extensive adhesions a left salpingectomy was not performed and a decision was made to proceed with clipping of the proximal part of the fallopian tube. The patient subsequently underwent in vitro fertilization (IVF) in a private fertility centre (2020) and five grade BB embryos were cryopreserved. Frozen embryo transfer (FET) of three of those (2020) failed to achieve a pregnancy, leaving two cryopreserved embryos available.

**Figure 1:**
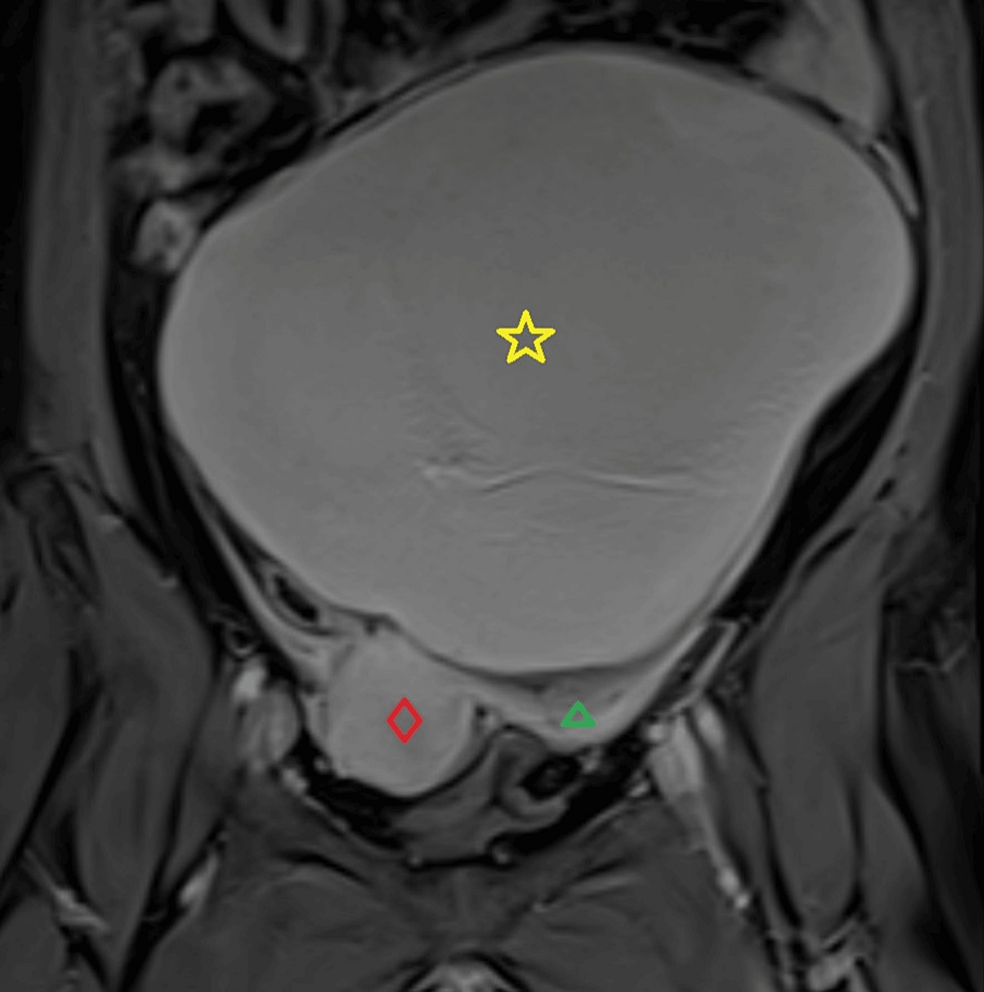
Preoperative MRI showing the left ovarian endometrioma (yellow star) with no features suspicious for malignancy, measuring 20.2 cm in maximal diameter, compressing the uterus (red rhombus) to the patient’s right side, and also showing the left hydrosalpinx (green triangle).

As part of the review, a transvaginal ultrasound scan (TVS) was performed and identified a large, unilocular ovarian cyst with ‘’ground-glass" echogenicity and colour score of 1 (minimal blood flow). The cyst fulfilled the criteria for a benign OMA, with no features suspicious for malignancy, occupying most of the pelvic cavity and measuring beyond 10 cm. As the entire OMA could not be confidently visualized by TVS and the patient was obese Class I (BMI=32), making transabdominal ultrasound (TAS) challenging to perform, a decision was made to proceed with magnetic resonance imaging (MRI) of the abdomen-pelvis. A large, thin-walled ovarian cyst was revealed, arising from the left ovary and measuring 20.2 cm in maximal diameter (up to the level of the patient’s umbilicus) with no solid components or features suspicious of malignancy (Figure 1).

The fluid-filled cyst exhibited high signal in T1 sequence with no loss of signal in T1 fat-suppressed sequence and low signal in T2 sequence. Features were typical of an OMA. The ovarian reserve was assessed preoperatively by measuring the level of anti-Mullerian hormone (AMH), which was 0.41 ng/mL. Ovarian tumour markers were also checked and the results were as follows: cancer antigen (CA) 125=151 U/ml, alpha-fetoprotein (AFP)=2.1 ng/ml, carcinoembryonic antigen (CEA)=0.6 ng/ml, and CA 19-9=16.4 U/ml.

**Figure 2:**
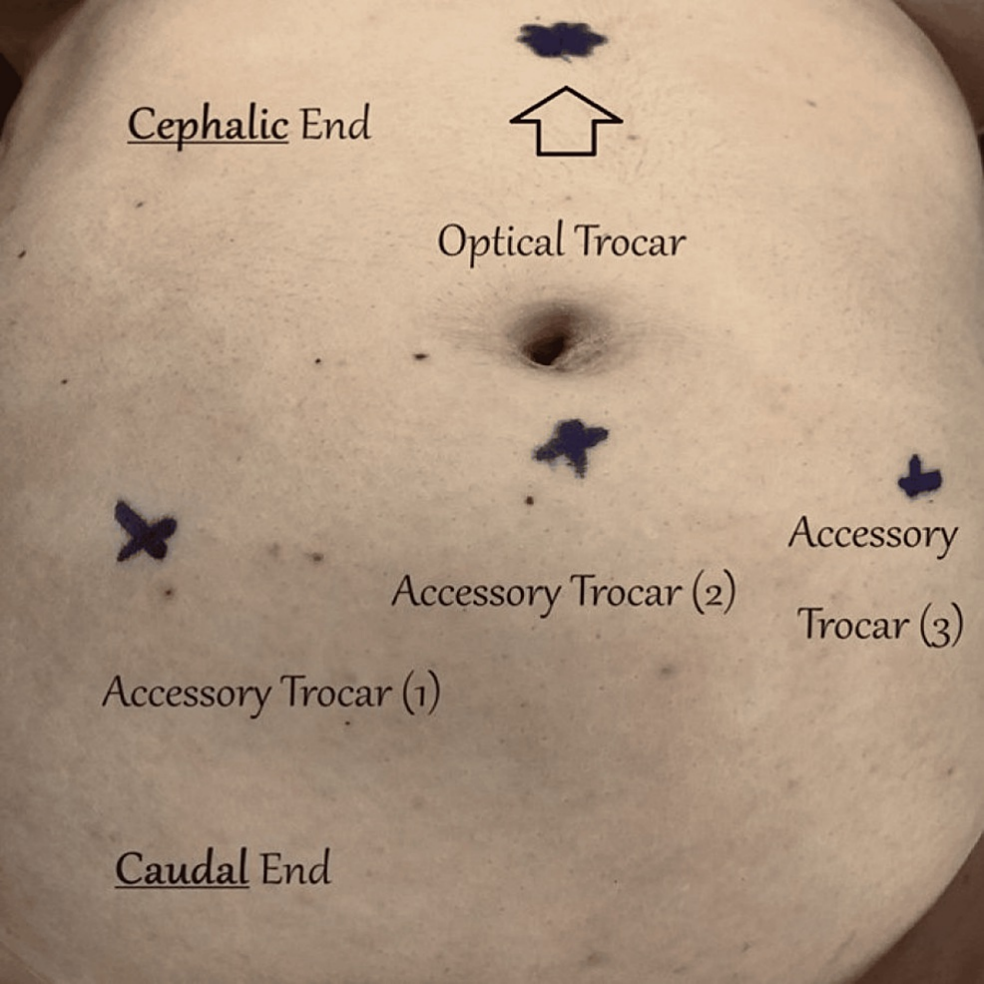
Marks on the patient’s anterior abdominal wall, just before the operation, reflecting the sites for primary and accessory trocar entry

As the patient was heavily symptomatic of the OMA, an informed decision was made to proceed with laparoscopic ovarian cystectomy. As the upper end of the OMA reached up to the umbilicus, insertion of the Veress needle in the Lee-Huang point (midway between the umbilicus and the xiphisternum) was preferred [8]. Initial intra-abdominal pressure of 7 mmHg was obtained and CO2 pneumoperitoneum was created uneventfully, in routine fashion, up to 20 mmHg. The pressure was maintained at 20 mmHg for the insertion of the primary, 12-mm port (laparoscopic camera) through the same incision, as well as three 5-mm accessory working ports inserted under direct vision, in positions slightly higher than usual, as seen in Figure 2.

**Figure 3:**
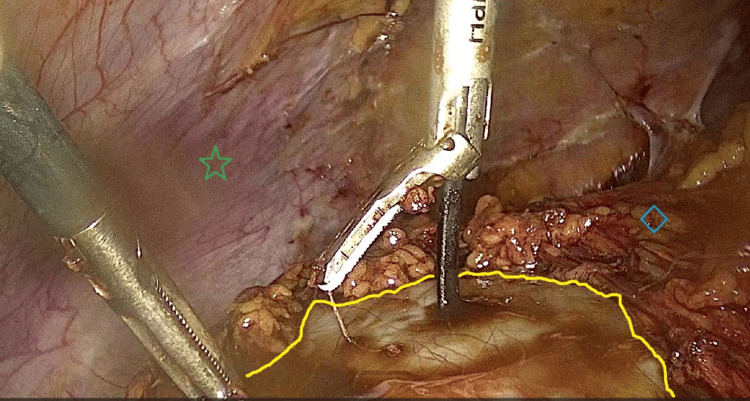
Intra-operative image of ovarian endometrioma A window was created on the endometrioma cyst wall (yellow line) to allow for a small incision, through which the laparoscopic suction-irrigation cannula was inserted. Note the close proximity between the large cyst and the abdominal wall (green star), as well as the omentum (blue rhombus) that is adherent to the cyst.

The voluminous cyst took up most of the operative field, limiting visibility. The omentum was adherent to the cyst wall and careful adhesiolysis was performed, with the cyst intact, to free the cyst wall from the omentum. It was then revealed that the sigmoid colon was also adherent to the anterior aspect of the cyst. Following adhesiolysis, a window free of adhesions was created and a small incision on the cyst wall was made (Figure 3), allowing insertion of the laparoscopic suction-irrigation cannula inside the cavity of the cyst and, hence, minimizing spillage of the chocolate cyst fluid in the abdominal cavity.

**Figure 4:**
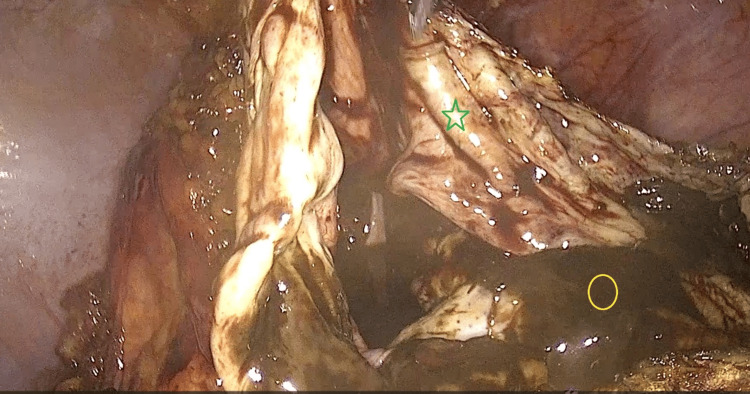
Intra-operative image of endometrioma cystectomy Following suction of the endometrioma content, the wall of the large cyst (green star) is exposed to the surgeon, prior to initiating the ovarian cystectomy. Note the characteristic chocolate fluid content (yellow circle) of the endometrioma.

Around 3.000 mm of chocolate cyst fluid was aspirated. Deflation of the cyst improved visualization and allowed the remaining adhesiolysis to be safely completed, using an ultrasonic surgical instrument (Harmonic scalpel™, Ethicon, Inc., Raritan, New Jersey, United States) until the OMA was completely free from adhesions. A plane of dissection between the OMA and the left ovarian cortex was identified and developed, leading to successful laparoscopic cystectomy (Figure 4).

Bipolar coagulation of bleeding points on the ovarian cortex was performed to achieve hemostasis. Suturing the remaining ovarian cortex was not deemed necessary. A 2-cm deficit in the meso-sigmoid was identified and the assistance of a colorectal surgeon was summoned. Inflating air and methylene blue in the recto-sigmoid revealed no evidence of inadvertent injury. A left salpingectomy was then performed in routine fashion. The incision for the left-sided 5-mm trocar was extended and a 10-mm port was inserted, through which a laparoscopic Endo Bag™(Medtronic plc, Dublin, Ireland) was used and the specimen was removed uneventfully. The rectus sheath of the 10-mm and 12-mm incisions was closed with Vicryl™ (Ethicon, Inc.), and the skin incisions with Monocryl™ (Ethicon, Inc.) sutures. Total blood loss was estimated at 30 ml and the procedure lasted 148 minutes.

**Figure 5:**
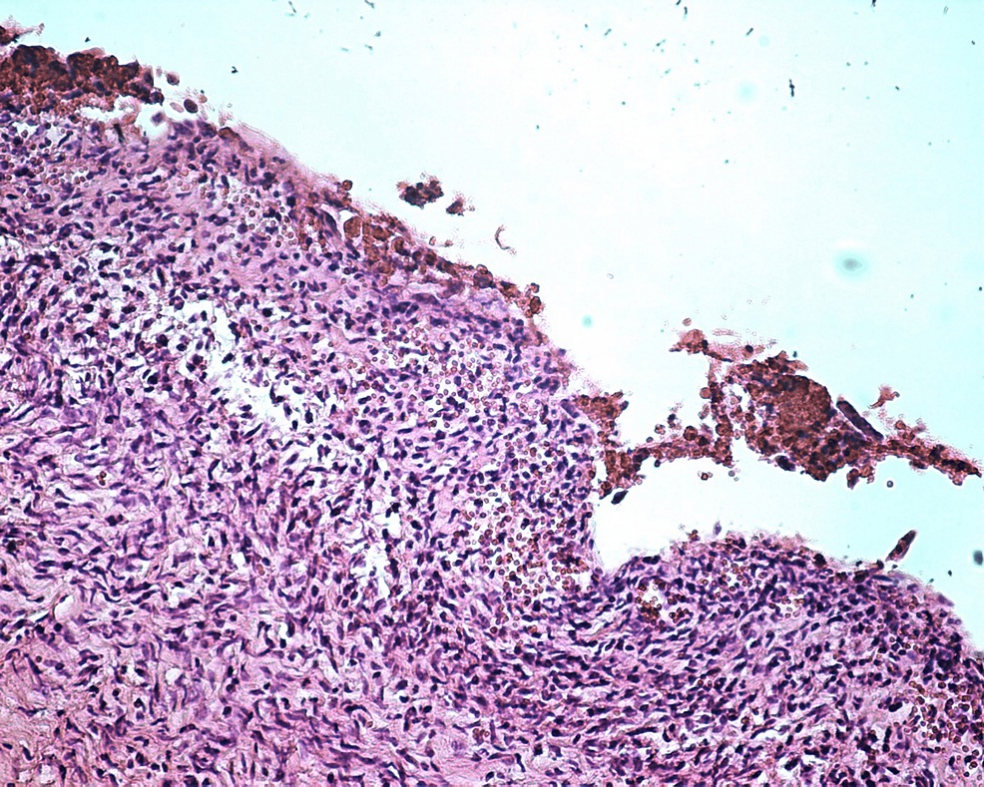
Microscopic examination of the endometrioma cyst wall revealed lack of surface epithelium with ferritin, macrophage cells, and hemorrhagic depositions, as well as presence of inflammation in the stroma

Haemoglobin dropped from 11.5 g/Dl to 10.6 g/Dl on postoperative day 1. The patient was discharged on postoperative day 2, after having opened her bowels, and made an uneventful recovery. A histological assessment of the specimen confirmed benign OMA (Figure 5).

## Discussion

This report describes the surgical management of a large OMA by laparoscopic cystectomy in a subfertile patient with previous surgeries and recurrent OMAs. Surgical management was anticipated to be challenging due to the very large size of the OMA as well as the patient’s past surgical history. Preoperative imaging strongly suggested that the nature of the cyst was benign, allowing us to safely employ a minimal access approach. Large, benign OMAs may also present for the first time in asymptomatic, postmenopausal patients and successful laparoscopic bilateral salpingo-oophorectomy has been described [8]. The largest OMA reported in the English literature was 65 cm in diameter [9].

As the upper end of the OMA extended up to the umbilicus, inserting the primary trocar in the routine fashion (base of the umbilicus) would have failed to provide an adequate view and would have risked inadvertent perforation of the OMA and potentially caused injury to other structures. Hence, we decided to utilize the Lee-Huang point [10], our commonly employed primary entry point in case of large pelvic pathologies, which provided satisfactory, panoramic views. Alternative options would have been the Palmer’s point [11], or the Jain point [12].

One option would have been to deflate the OMA right at the start of the procedure after having inserted the trocars. However, in the reported case, the omentum and the sigmoid colon were adherent to the OMA. Blindly perforating the OMA could have caused haemorrhage due to injury to the vascular omentum or perforation of the sigmoid colon. In contrast, we decided to perform adhesiolysis with the OMA intact, aiming to identify a safe window on the cyst wall to make an incision large enough to just insert the laparoscopic suction-irrigation cannula. Creating a larger incision on the cyst wall from the start would have caused diffuse spilling of the chocolate cyst fluid, staining the operative field and hampering the surgeon’s vision. However, we acknowledge that completely preventing the spillage of OMA content is not feasible, even when employing this approach.

A recent study suggested that the presence of living endometrial cells in ovarian endometriotic cyst fluid may contribute to the recurrence of endometriosis after surgical excision of OMAs [13]. We recommend that adhesiolysis (which requires excellent vision) is more easily performed with the OMA intact (rather than deflated); however, the risk of rupturing the pathology during adhesiolysis needs to be balanced. Identifying the plane of dissection between the OMA and the ovarian cortex may be particularly challenging in case of large OMAs and significant surgical experience is required. There is evidence that the degree of ovarian damage during OMA cystectomy is proportional to the OMA size [14].

Our patient had very low ovarian reserve, as assessed by pre-operative AMH levels. This was anticipated due to previous surgeries to the ovaries [15], as well as the presence of endometrioma per se [3], although recent evidence suggests that AMH may be unexpectedly high in the case of large OMAs [16]. The low AMH level raises the question of whether alternative, non-excisional techniques of OMA surgery could have been employed. Ablative techniques appear to cause less damage to the healthy ovarian cortex [17]; however, ablating such a large surface would not have been, in our view, feasible.

Alternative approaches were discussed with the patient and included laparoscopic ethanol sclerotherapy [18], of which we have not yet had any experience in performing, and ‘’three-step’’ procedures [19,20]. As regards the latter, we feel that simple fenestration and drainage of the OMA during the first surgery may not have been successful in providing adequate and sustained symptomatic relief to the patient. It should also be noted that the patient was managed privately (the cost of repeat surgeries was, therefore, taken into consideration) and there existed two cryopreserved embryos. Prioritization was, therefore, given to managing the patient’s severe symptoms; hence a decision was made to employ a more radical approach (cystectomy) with an aim to also reduce the risk of recurrence.

## Conclusions

Large OMAs can be safely managed by laparoscopic cystectomy by surgeons with appropriate expertise, provided that certain technical rules are respected. Adequate preoperative imaging can reliably exclude ovarian malignancy, allowing for the safe application of minimal access techniques. Non-excisional techniques may be employed as alternatives to OMA cystectomy in selected cases; however, they may be less appropriate in severely symptomatic patients. There is no reliable way to prevent OMA development but routine gynecological sonographic surveillance of high-risk groups may allow earlier diagnosis and intervention, either medical or surgical.
